# Oxetane Synthesis through the Paternò-Büchi Reaction

**DOI:** 10.3390/molecules180911384

**Published:** 2013-09-16

**Authors:** Maurizio D’Auria, Rocco Racioppi

**Affiliations:** Dipatimento di Scienze, Università della Basilicata, Viale dell'Ateneo Lucano 19, 85100 Potenza, Italy; E-Mail: rocco.racioppi@unibas.it

**Keywords:** photochemistry, (2+2)-cycloaddition, oxetanes, organic synthesis

## Abstract

The Paternò-Büchi reaction is a photochemical reaction between a carbonyl compound and an alkene to give the corresponding oxetane. In this review the mechanism of the reaction is discussed. On this basis the described use in the reaction with electron rich alkenes (enolethers, enol esters, enol silyl ethers, enanines, heterocyclic compounds has been reported. The stereochemical behavior of the reaction is particularly stressed. We pointed out the reported applications of this reaction to the synthesis of naturally occuring compounds.

## 1. Introduction

**Figure 1:**
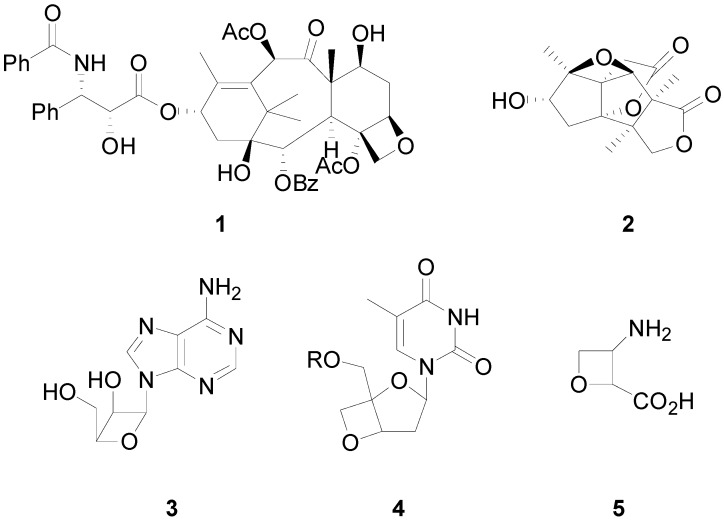
Natural and biologically active compounds containing the oxetane ring.

**Scheme 1:**
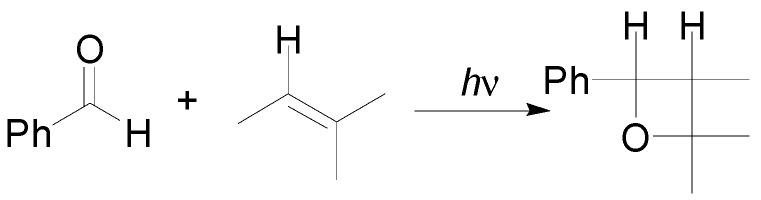
The reaction performed by Paternò.

Oxetanes are important component in the scaffold of compounds with relevant biological properties: an oxetane ring is present in the scaffold of Taxol^®^ (**1**), an important drug used in the treatment of ovarian cancer [1], merrilactone A (**2**), a sesquiterpene dilactone with neurotrophic activity [2], and several antiviral oxetanes, such as **3**, **4**, and **5**, have been described in the literature (Figure 1) [3,4,5]. In 1909 Paternò, studying the photochemical reaction of benzophenone with amylene, showed the formation of the corresponding [2+2]cycloadduct (Scheme 1) [6].

Only in 1953 Büchi reported the exact identification of the product, showing that this photochemical reaction could represent an interesting synthetic process [7]. After these pioneering works, several reviews have covered the relevant papers published in this field [8,9,10,11,12,13,14,15,16,17,18,19,20,21,22,23,24].

**Scheme 2:**
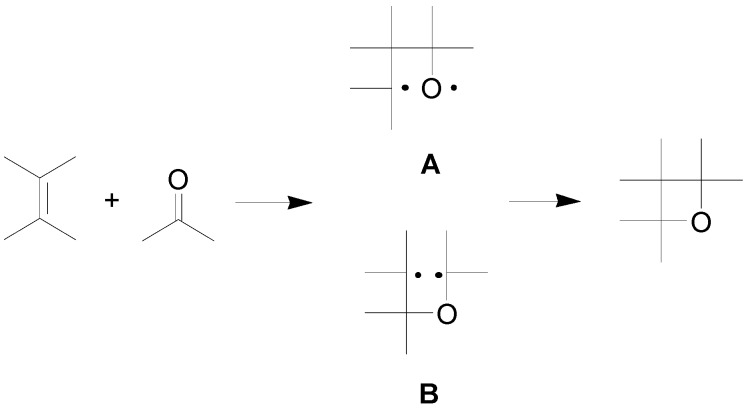
Possible mechanisms of the Paternò-Büchi reaction.

The reaction is a photocycloaddition reaction of a carbonyl compound in the excited state with an alkene in the ground state. The carbonyl compound must posses a n,π* S_1_ or T_1_ excited states. The frontier orbitals approach has been used to explain the formation of oxetanes. HSOMO-LUMO interaction is the main process observed where the half-occupied π* orbital of the carbonyl compound interacts with the unoccupied π* molecular orbital of an electron deficient alkene: the results of this interaction is the formation of a C,O-biradical (**A** in Scheme 2). If the LSOMO-HOMO interaction is prevalent (interaction of the half-occupied n orbital of the carbonyl O atom with the π orbital of an electron-rich alkene), the formation of a C,C-biradical (**B** in Scheme 2) is the main process [25,26].

The majority of Paternò-Büchi reactions occur from the carbonyl triplet state which is accessed by an intersystem crossing. The biradicals are the direct consequence of the precursor spin and their lifetimes are connected with the mode of spin inversion processes and the mechanism, which leads to the formation of closed-shell products. 1,4-Biradicals derived from the addition of a carbonyl compound in its first excited triplet state and an alkene were studied spectroscopically [27,28,29,30] and they were trapped by radical quenchers [31,32,33,34]. The biradical intermediate in the reaction between benzophenone and an electron-rich alkene has been determined by using laser flash photolysis. An absorption with λ_max_ 535 nm has been observed [27,29].

In the reaction between 1,4-dioxene and benzaldehyde, theoretical calculations showed that the only transition able to give the observed transient absorption is that from LSOMO to LUMO (549 nm); the same result was obtained for the reaction between furan and benzaldehyde [21].

The regioselectivity of the reaction can be explained considering the hard-soft acids and bases theory [35], or using an approach where atoms arrange themselves so that the obtained molecule reaches the minimum electrophilicity [36].

A theoretical study of the Paternò-Büchi reaction showed that there are two conical intersection points located near the C-C and C-O bonded biradical regions of the ground state. These two conical intersections support a mechanism where the decay from the excited state is accompanied by a geometric rotation of the terminal group, in the case of C-O attack, and by an orbital rotation at the oxygen center, in the case of C-C attack. Thus, for the triplet, the reaction path can be predicted by the most stable biradical rule [37]. A conformational analysis of the biradicals also appeared [38,39].

A CAS SCF geometry optimization by using TZV basis set of the intermediate biradicals showed that the diradical region corresponding to the C-C attack lies about 10 kcal mol^-1^ lower in energy than the C-O region [40,41]. Unfortunately, this result is not in agreement with reported experimental results.

The formation of an exciplex is used to explain the reaction behavior of simple alkene. Evidence of monoelectron transfer processes is reported for electron-rich alkenes [42].

## 2. How to Perform the Reaction

The Paternò-Büchi reaction can be performed in solution. The effect of the solvent is relevant and non-polar solvents are preferred. When an aromatic carbonyl compound is used as substrate the irradiation has to be performed at 300 nm through Pyrex, while the use of aliphatic carbonyl compounds needs the use of 254 nm irradiation through quartz or Vycor. The quantum yields of the reaction are not high. The photochemical coupling of benzophenone, or another carbonyl compound, with itself to five the corresponding pinacone derivative is a competitive reaction allowing to give low quantum yields (typically, 10^−1^–10^−2^).

## 3. Reactions with Electron Rich Unsaturated Compounds

The first oxetane deriving from ethyl vinyl ether and benzophenone was described by Paternò [43]. He described the formation of the oxetane in the reaction of benzophenone with diethyl ether. He suggested the *in situ* formation of ethyl vinyl ether. The reaction between acetone and ethyl vinyl ether in the presence of ultrasound gave a different mixture of regioisomers than that obtained without the use of ultrasound [44].

**Scheme 3:**
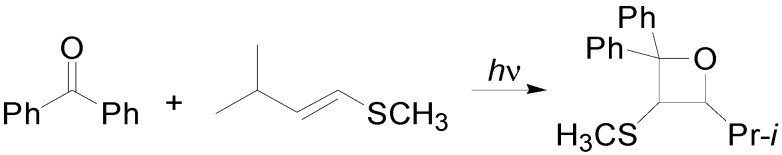
Reaction of benzophenone with alkenyl sulphide.

A high regioselectivity was obtained when ethyl vinyl ether was used as an alkene with benzaldehyde and benzophenone [45]. The reaction of benzophenone with alkenyl sulphide also showed a high regioselectivity (Scheme 3) [46].

Substituted pyruvates reacted with 1,3-dioxole and this reaction allows one to obtain the *endo* isomer [47,48,49]. The analysis of the conformations of the biradical intermediate was used in order to explain the observed stereochemical behavior [50]. Unsubstituted cyclopropyl enol ethers gave, when reacted with a carbonyl compound, the corresponding oxetane rings. When a substituent is introduced on the cyclopropyl ring, the formation of oxepanes was observed deriving from cyclopropyl ring opening at the level of the biradical intermediate [51].

**Scheme 4:**
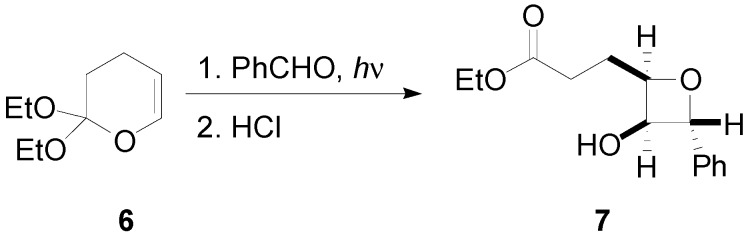
Reaction of an enol ether.

When the enol ether **6** was irradiated in the presence of benzaldehyde, and the reaction mixture was purified after an acid hydrolysis, the oxetane **7** was isolated in good yields with good diastereoselectivity (Scheme 4) [52].

**Scheme 5:**
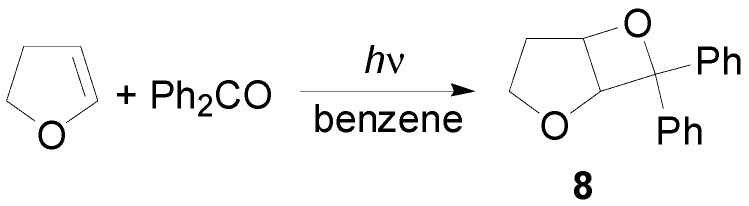
Reaction of 2,3-dihydrofuran.

1,2,3-Indanetrione reacted with 2,3-diphenyl-1,4-dioxene to give the oxetane on the carbonyl in the 2 position [53]. On the contrary, when the less electron rich 2,3-dimethyl-2-butene is used as alkene, a complex mixture of products is obtained [54]. The irradiation of 2,3-dihydrofuran with benzophenone in benzene gave adduct **8** (Scheme 5) [55,56,57].

The selectivity depended on 2,3-dihydrofuran concentration: this behavior was explained with a switch from a triplet mechanism to a singlet mechanism at higher concentration [58,59,60,61,62]. This behavior can be explained [63]. The best interaction between the frontier orbitals is that from the LSOMO of acetaldehyde and the HOMO of 2,3-dihydrofuran. The atomic coefficients on the olefinic carbon atoms in 2,3-dihydrofuran were -0.26 at C-2 and -0.38 at C-3. The atomic coefficient on the oxygen atom in the LSOMO of singlet excited acetaldehyde was 0.48, while the atomic coefficient at the C-1 of acetaldehyde in the HSOMO was 0.49.

**Scheme 6:**
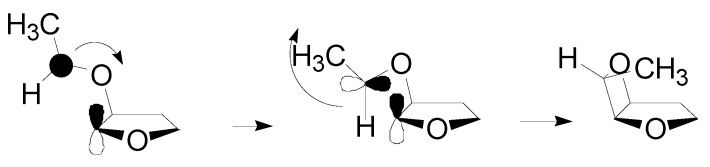
Ring closure of the oxetane ring in the reaction of 2,3-dihydrofuran with acetaldehyde.

The nature of the LUMO of 2,3-dihydrofuran excludes the possibility of a concerted mechanism. The reaction has to provide for the formation of extremely labile singlet biradical. In this case, the oxygen atom of acetaldehyde has to attack the C-3 carbon atom in 2,3-dihydrofuran in order to give the more stable biradical intermediate. The reaction, in this case, allowed the formation of only the *exo* isomer. In the triplet state, the main interaction is that between the LSOMO of the triplet state acetaldehyde and the HOMO of the dihydrofuran. This interaction leads to the formation of the corresponding CC biradical intermediate (Scheme 6).

The HSOMO on the biradical intermediate was mainly localized on the aromatic ring and it is extended to the radical carbon. The LSOMO was mainly localized on the dihydrofuran ring. The coupling between the radical carbons in these two orbitals was possible (the atomic orbitals involved can superimpose themselves) only if the *endo* isomer was formed (Scheme 6).

When benzaldehyde was used as carbonyl compound, the reaction showed a good regio- and stereoselectivity [61,64,65,66]. The adducts were obtained with an overall yield of 98% as a > 98:2 regioisomeric mixture; the major isomer is 88:12 *endo/exo* mixture. The stereoselectivity was partially lost when benzaldehyde reacted with a 2,3-dihydrofuran derivative substituted in the 2 position [64,67].

A change of regioselectivity was observed when the reaction between 2,3-dihydrofuran and benzaldehyde was performed in high polar solvent: this result has been interpreted as proof of an electron transfer mechanism [58,59].

The reaction of dihydrofuran with benzaldehyde is the first example where a spin controlled selectivity is observed [60,68]. In singlet photoreactions, stereoselectivity is often controlled by the optimal geometries for radical-radical combinations. On the contrary, in triplet photoreactions the optimal geometries are those able to favor the intersystem crossing from the triplet excited state to the singlet one. The singlet biradicals should be too short-lived to enable rotation about the endocyclic C-O or C-C bonds and therefore, conformation memory effects on the stereochemistry are expected. The geometries in the triplet state can be quite different from the former ones due to differences of the spin-orbit coupling (SOC) values. The lifetimes of many triplet biradical intermediates are definitely high enough to enable bond rotations. Therefore, the formation of the thermodynamically favored product can be expected because the radical-radical combination step should not be influenced by the approach geometry: “memory effects” should be erased due to the relatively long lifetimes. After transition from the triplet to the singlet potential energy surface, immediate product formation is expected. Thus, the ISC proceeds in a concerted fashion with the formation of a new bond or the cleavage of the primarily formed single bond. As a consequence, the stereoselectivity of the Paternò-Büchi reaction is the result of a combination of several rate constants for cyclization versus cleavage reactions.

**Scheme 7:**
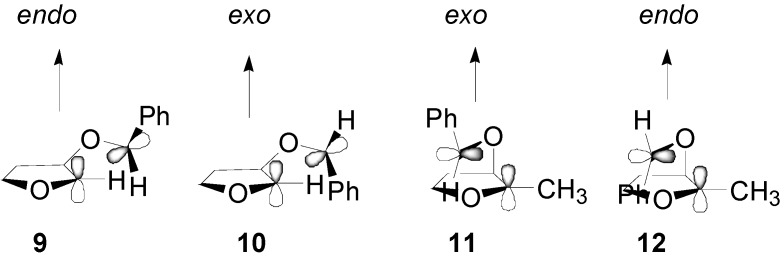
Possible biradicals in the reactions between 2,3-dihydrofuran and benzaldehyde.

Benzaldehyde reacts in its triplet state. This way, a triplet biradical intermediate is formed. To obtain the products, intersystem crossing into the singlet manifold is necessary. The most important factor influencing an intersystem crossing for flexibile triplet biradicals is spin-orbit coupling. The angle between *p* orbitals at the radical centers is approximately 90° for maximun spin orbit coupling. For the pronounced *endo* selectivity in the reaction between aromatic aldehydes and 2,3-dihydrofuran, we can consider the two biradical conformers **9** and **10** to be responsible, with the alkyloxy substitutent localized in a pseudoequatorial position and **9** being more populated because of fewer steric interactions (Scheme 7).

**Figure 2:**
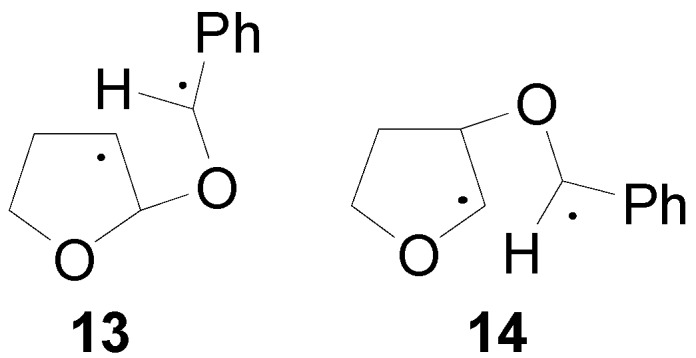
Possible biradical intermediates in the reaction of 2,3-dihydrofuran with benzaldehyde.

When a methyl group is present, the increasing *gauche* interactions with the β-alkyloxy substituent lead to a certain concentration of **11** and **12**, again with **11** being preferred because of fewer steric interactions. Another explanation for the regio- and stereochemistry appeared [69]. In fact, theoretical calculations showed that the biradical **13** is more stable than **14** by 1.49 kcal mol^−1^ (Figure 2).

**Scheme 8:**
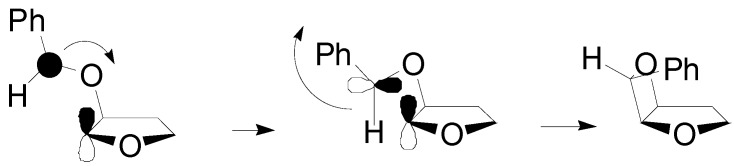
Ring closure reaction in the formation of the *endo* isomer of the adduct between 2,3-dihydrofuran and benzaldehyde.

Furthermore, the HSOMO was mainly localized on the aromatic ring and it is extended to the radical carbon. The LSOMO was mainly localized on the dihydrofuran ring. The coupling between the radical carbons in these two orbitals was possible (the atomic orbitals involved can superimpose themselves) only if the *endo* isomer was formed (Scheme 8) [69].

**Scheme 9:**
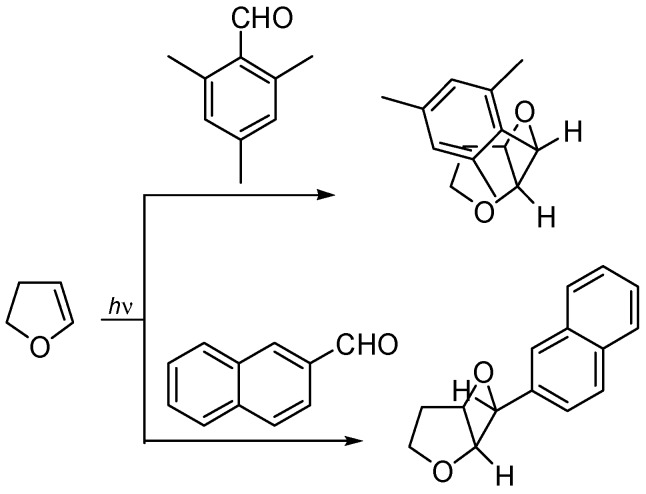
Different stereochemical behavior in the reactions of 2,3-dihydrofuran.

α- and β-Naphthaldehydes, on the contrary, gave high *exo* selectivity (Scheme 9). The reaction occurred also in the presence of triplet quenchers, while fluorescence quenching in the presence of dihydrofuran was observed.

In this case, the singlet excited state was responsible for the high *exo* selectivity [61,70]. In this case, the coefficients on the HSOMO and LSOMO allowed the coupling of the radical carbons only if the *exo* isomer is obtained [71].

When 2,3-dihydrofuran derivatives react with α,β-unsaturated carbonyl compounds, 2+2 cycloaddition between the olefins occurs [72]. Benzaldehyde reacts with L-ascorbic acid giving a mixture of regioisomeric compounds with *exo* stereoselectivity [73].

**Scheme 10:**
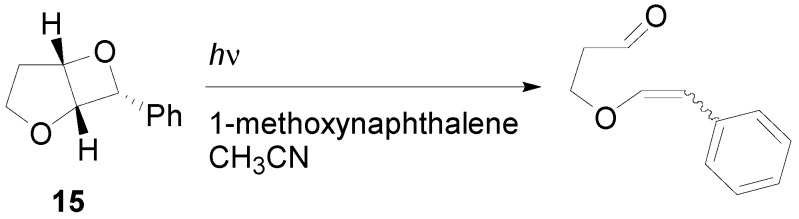
Metathesis reaction on an oxetane.

Irradiation of the oxetane **15** in the presence of 1-methoxynaphthalene and 2,7-dimethoxynaphthalene as sensitizers gave an efficient carbonyl-alkene metathesis of bicyclic oxetanes through an electron transfer process (Scheme 10) [74].

**Scheme 11:**
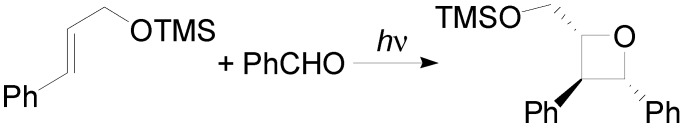
Reaction of a silyl derivative of cinnamyl alcohol.

A cycloreversion reaction was supposed also in the photochemical reaction of α,α-diphenyl substituted diazotetrahydrofuranones [75]. High regio- and stereoselectivity (*syn* addition) was observed in the reaction of pentafluorobenzaldehyde with enol acetates [76]. The reaction between a 2,3-thiophenone derivative and vinyl acetate gave the corresponding adduct on a ketonic function in low yields [77]. The reaction between benzaldehyde and a silyl derivative of cinnamyl alcohol gave the corresponding oxetane with high stereoselectivity (Scheme 11) [78].

**Scheme 12:**
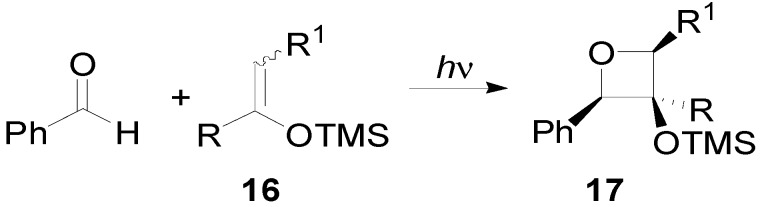
Reaction of silyl enol ether.

3-(Silyloxy)oxetanes **17** were successfully prepared from silyl enol ethers **16** containing carbon-chlorine, carbon-silicon, or carbon-sulfur bonds (Scheme 12) [79,80,81]. Ether and ester groups were compatible with the reaction. The presence of an alkene moiety was also compatible. When a β-alkyl substituted silyl enol ether was used, a *trans* relationship between α and β susbstituents in the oxetanes was observed. This result did not depend on the (*E*)- or (*Z*)- nature of the alkene. The products were obtained with high simple diastereoselectivity (ds 74%–95%) [82].

In the triplet biradical, free rotation leads to the highly preferred, sterically least congested conformation. The further reaction pathway of this species includes ISC and an assumed selection step (cleavage vs. ring closure) at the singlet 1,4-diradical level which accounts for the high simple diastereoselectivity at C-2/C-3.

The oxetanes can be converted into the corresponding diols by using hydrogenolysis under Pd catalysis. Acid-sensitive substrates can be hydrogenated using Pd(OH)_2_ as catalyst [83]. Also LiAlH_4_ can be used in order to induce the cleavage of the oxetane ring [84].

**Scheme 13:**
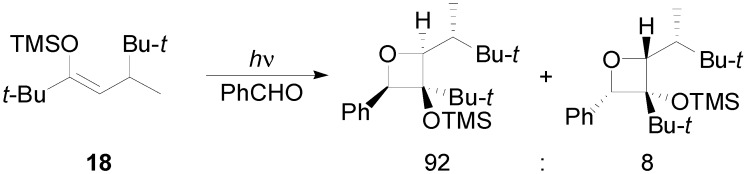
Diastereoselectivity in the reaction of a silyl enol ether.

The presence of a stereogenic center in the β-alkyl group (as in **18**) induced a facial diastereoselectivity. In some cases, high diastereoisomeric ratios were observed (Scheme 13) [85].

**Scheme 14:**
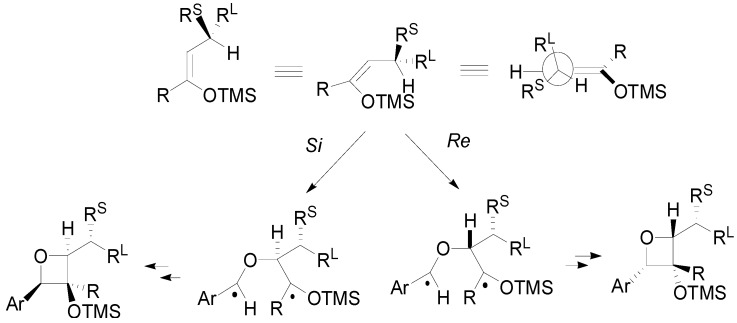
Diastereoselectivity in chiral enol silyl ethers reaction with benzaldehyde.

The diastereoselectivity was probably due to the presence of a conformational preference represented in the Scheme 14. This conformation allows the *Si* attack [84].

**Scheme 15:**
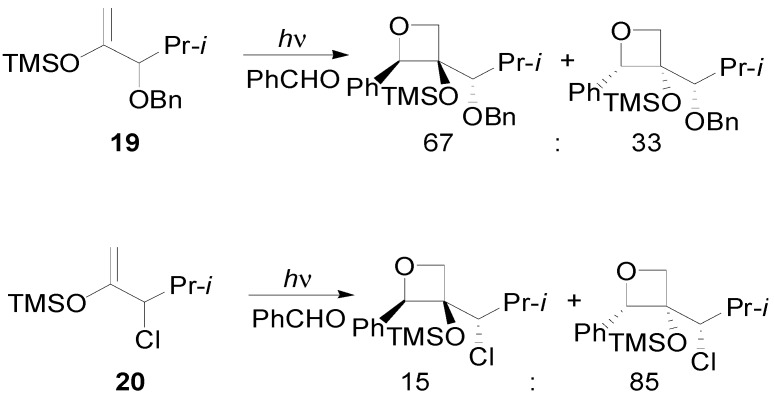
Diastereoslectivity on the reaction of some enol ethers.

In this case, a fluoride-promoted cleavage can be used to open the oxetane ring [85]. When the chiral center is in α position low facial diastereoselectivity was observed [86]. The best results were obtained by using both the silyl enol ether **19**, which gave the adducts with a 67/33 d.r., and the compound **20**, giving the adducts in 15/85 d.r. (Scheme 15) [87].

**Figure 3:**
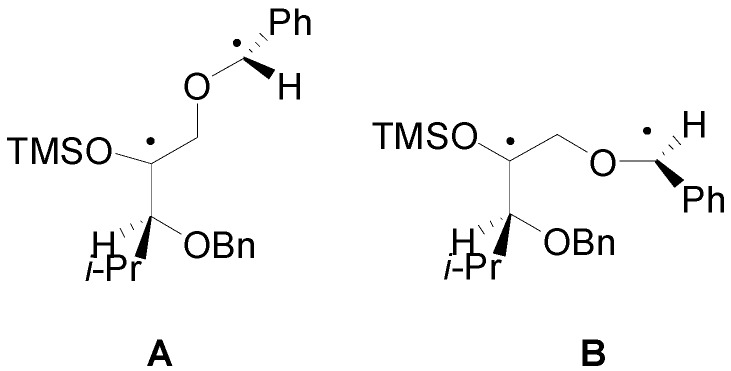
The conformers (**A** and **B**) of the biradical intermediate in the photoreaction between **19** and benzaldehyde.

In the reaction of **19** with benzaldehyde two conformers of the biradical intermediate have been observed. These conformers showed almost the same energy (the conformer **A** is more stable than **B** for 3.11 kcal/mol): the conformers of the biradical are shown in Figure 3 [70].

**Scheme 16:**
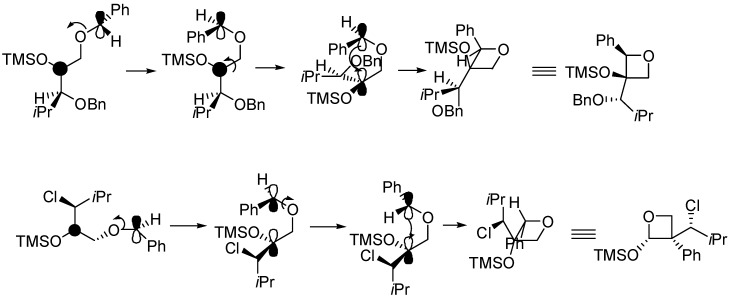
Possible explanation of the observed diastereoselectivity in the reaction of chiral enol ethers.

Considering the reactive sites and the atomic coefficients at these sites, the coupling of the radical carbon atoms can occur only as depicted in the Scheme 16.

The conformer **A** can give the major stereoisomer observed in the reaction. The results are in agreement with the experimental results showing that the course of this reaction is strictly frontier orbitals controlled. The other biradical conformer gave the other diastereoisomer. The observed diastereoisomer ratio (67:33) can be explained by the low difference between the energies of the conformers of the biradical intermediate (3.11 kcal/mol).

To confirm this result the behavior of **20** has been examined. In this case, the authors observed an inverse diastereoselectivity [86]. Also in this case two conformers of the biradical intermediate were possible. The energy difference between these two conformers was 4.6 kcal/mol. The coupling between the carbon atom considering the atomic coefficents allowed the formation of the observed diastereoisomers: the most stable conformer gave the main product. The larger diastereoisomeric ratio (85:15) here observed in comparison with that found in the other case is in agreement with the larger energy difference between the conformers of the biradical intermediate.

**Scheme 17:**
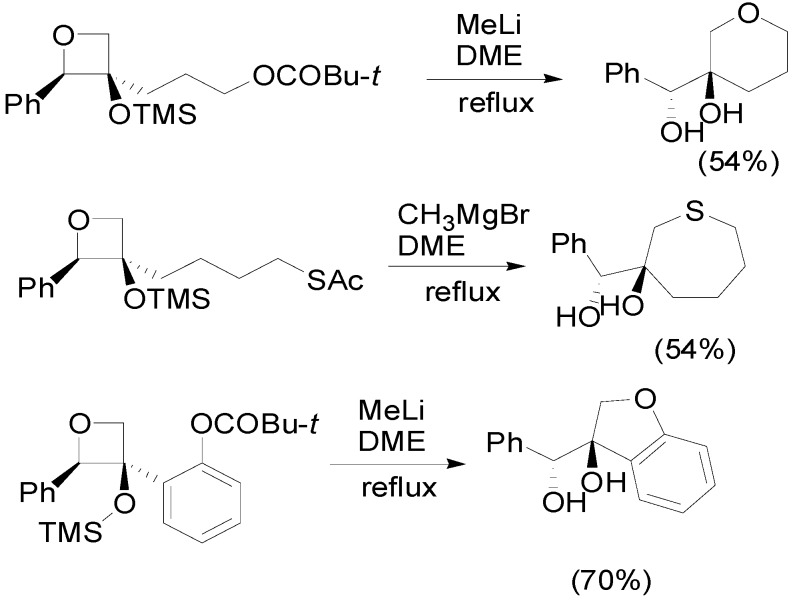
Elaboration on the oxetane ring.

The synthetic utility of the oxetanes has been studied considering the presence of a removable protecting group in the side chain of the same oxetane. The possible intramolecular nucleophilic attack on the oxetane ring gave some interesting products (Scheme 17) [87].

1,2-Diketones reacted with trimethylsilyl ketene acetals giving the corresponding oxetanes in low yields [88]. In the reaction of ketene silyl acetals with aromatic carbonyl compounds byproducts have been identified as oxetanes: in some cases they represent the main product [89]. Cyclic ketene silyl acetals reacted with 2-naphthaldehyde to give the corresponding adduct. The treatment of the reaction mixture with water gave the aldol-type product with high stereoselectivity. The oxetane was obtained with *anti* stereochemistry [90,91]. The effect of both the solvent and the nature of the silyl group has been examined [92]. When silyl O,S-ketene acetals were used as alkene some interesting results were obtained [93].

**Scheme 18:**
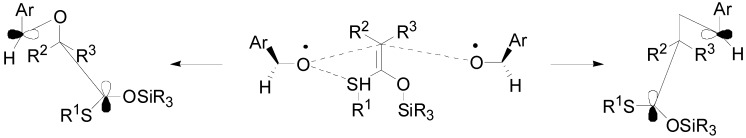
The reaction O,S-ketene acetals with carbonyl compound. A possible explanation of the stereochemical behavior.

The stereochemical behavior was explained by considering the capability of the sulfur atom to coordinate the oxygen atom of the carbonyl compound [93]. The presence of such an atom induced the attack of the carbonyl compound on the side where the sulfur atom is present (Scheme 18). The same regio-and stereoselectivity was observed when silyl O,Se-ketene acetals were used [94].

**Scheme 19:**
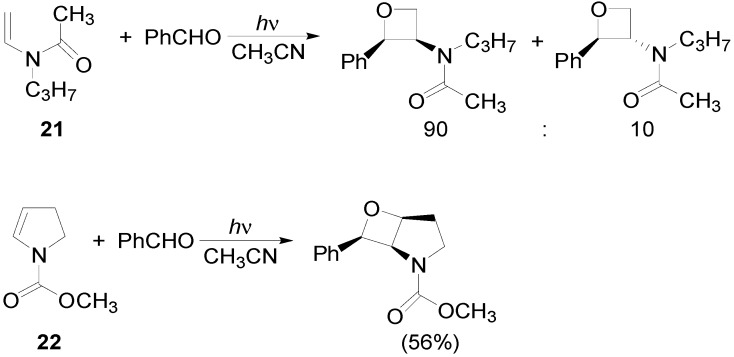
Reacions of *N*-acyl enamines.

Oxetanes were obtained in the reaction of *N*-acyl enamines **21** and **22**, these compounds gave the corresponding adducts with high regio- and stereoselectivity (Scheme 19) [95,96,97,98]. The main product was the thermodynamically more stable isomer [99].

**Figure 4:**
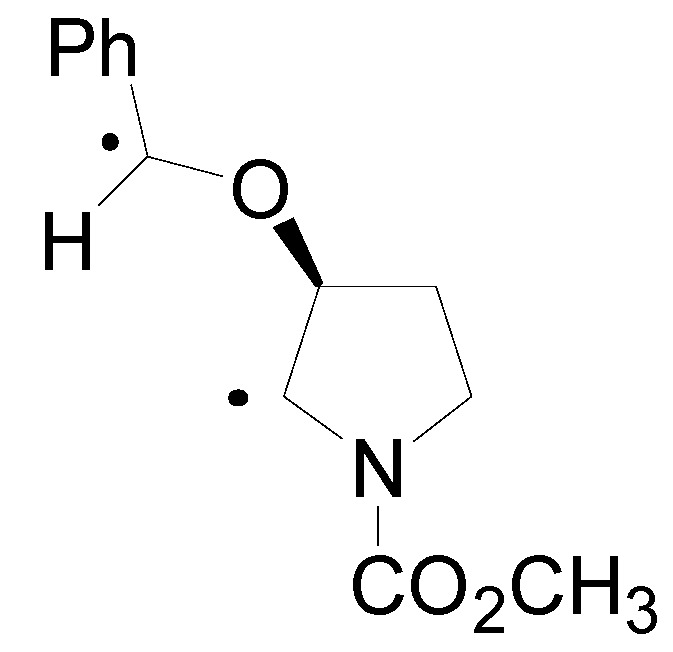
Optimized structure of the biradical intermediate in the reaction of compound **22**.

The optimized structure of the biradical intermediate is shown in Figure 4 with the HSOMO at −0.215 H and the LSOMO at −0.241 H [70].

**Scheme 20:**
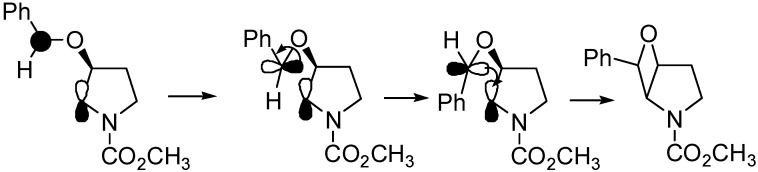
Possible explanation of the stereoselctivity observed with *N*-acyl enamines.

The coupling of the radical carbon atoms considering the atomic coefficients on the SOMOs allowed the formation of the *exo* isomer, in agreement with the experimental results (Scheme 20).

**Scheme 21:**
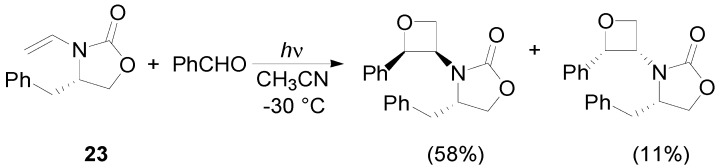
Reaction with a chiral enamine.

Chiral enamines did not give the corresponding adduct with high diastereoselectivity. The only exception was found using the enamine **23**. It gave the corresponding adduct with 62% *de* (Scheme 21) [100,101].

*N*-Formyl protected oxetanes could be converted into the corresponding *syn*-1,2-amino alcohols through treatment with LiAlH_4_ [97]. Alternatively, the treatment with TFA gave the corresponding oxazolidinone. Furthermore, the product can be converted into the corresponding *anti*-1,2-amino alcohol [97,102]. This approach has been used in the synthesis of both (±)-oxetin [103] and (+)-preussin [104,105].

The Paternò-Büchi reaction could be obtained also using alkenes bearing both electron donating groups (nitrogen atoms) and electron withdrawing groups. 2-Morpholinopropenenitrile gave in very low yields a product deriving from a Paternò-Büchi reaction when it reacted with naphthalene-1,4-dicarboxylic acid [106]. The same result was obtained when benzil was used as substrate. On the contrary, when methyl phenylglyoxylate was used as carbonyl compound, the corresponding adduct was isolated in good yields [107].

When benzil was irradiated in the presence of a 2-aminopropenenitrile derivative gave the corresponding adduct in variable yields [108]. The reaction showed a good stereoselectivity. When unsymmetrical benzil derivatives are used, both the isomers were obtained.

## 4. Reactions with Heterocyclic Compounds

The reactivity of pentatomic aromatic heterocycles different from furan towards carbonyl compounds to give the corresponding oxetanes has been the object of other review articles [109]. These compounds showed lower reactivity than furan (see below). The reason of this behavior is not clear. It could be related to the different aromaticity of these compounds in comparison with that of furan, or could be due, as reported below, to the quenching properties of the heterocycles.

**Scheme 22:**
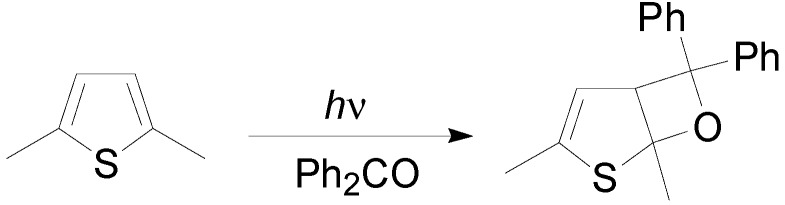
The reaction of 2,5-dimethylthiophene with benzophenone.

Thiophene did not react with benzophenone. The reaction occurred only if the irradiation is performed in the presence of BF_3_ [110]. Probably, in this reaction BF_3_ was able to catalyze the ring opening of the oxetane. The benzophenone BF_3_ complex, excited by light, leads to an exciplex whose excitation energy was lower that the lowest triplet energy level of thiophene, which under the circumstances cannot act as a quencher. On the contrary, 2,5-dimethylthiophene reacted with benzophenone at −10 °C yielding the corresponding cycloadduct in 62% yield (Scheme 22) [111,112].

The reaction product could be obtained also using 1-naphthaldehyde (50%), 2-, 3-, and 4-benzoyl- pyridine (62, 58, and 60%, respectively), and 2-benzoylthiophene (50%), while 2-naphthaldehyde, benzaldehyde, and acetophenone did not react [113]. 2,3-Dimethylthiophene also gave the corresponding oxetane when irradiated in the presence of benzophenone (60%) [114]. On the contrary, 2,3-dimethyl- and 2,3,5-trimethylthiophene did not react.

**Scheme 23:**
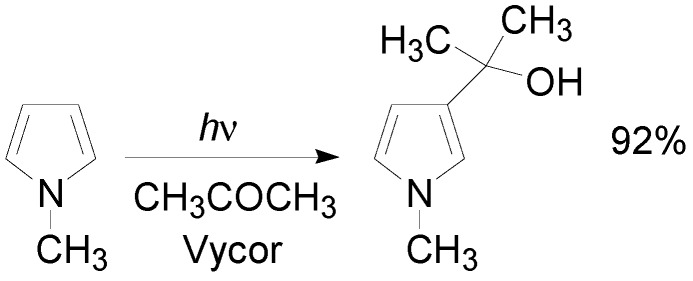
The reaction of *N*-methylpyrrole with acetone.

Pyrrole, such as thiophene, did not react with benzophenone and did not furnish the corresponding oxetane. However, pyrrole reacted with aliphatic aldehydes and ketones giving the corresponding 3-pyrryl carbinols. The alcohols derived from the cleavage of the corresponding oxetanes (Scheme 23) [115]. The yields increased when *N*-methylpyrrole was used as substrate, while the reactivity was depressed in the presence of subsituents on the pyrrole ring.

Only when pyrrole, with an electron withdrawing group such as benzoyl on to the nitrogen atom, was irradiated in the presence of benzophenone, it was able to give the corresponding oxetane [116,117]. When *N*-phenylpyrrole was used as substrate, the corresponding 2-pyrryl carbinol was isolated [116].

Selenophene did not react with benzophenone [111]. On the contrary, the irradiation of 2-methylselenophene gave the corresponding adduct. The reaction occurred on the most hindered side of the molecule [118].

**Scheme 24:**
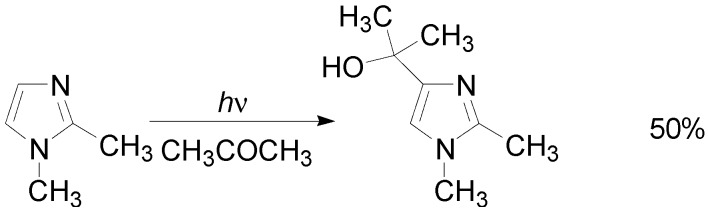
Reaction of *N*-methyl-2-methylimidazole with acetone.

Imidazole, *N*-methylimidazole, and 1,2-dimethylimidazole reacted with aliphatic aldehydes and ketones. In all the cases, the product was the corresponding 4-imidazolyl carbinols (Scheme 24) [114,119]. On the contrary, the reaction of imidazole with benzophenone gave only 7.7% yield of the corresponding alcohol [119], while, the same reaction, performed in the presence of acetophenone as carbonyl compound, gave only 1.2% yield of the product [119].

**Scheme 25:**
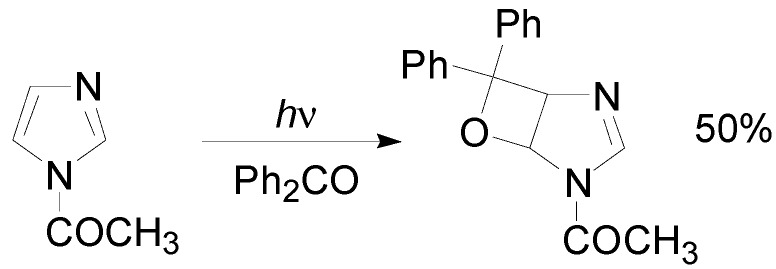
The reaction of *N*-acetylimidazole with benzophenone.

On the photochemical behavior of *N*-methylimidazole, there was not agreement between the work of Jones [115] and that of Matsuura [119]. While Jones reported that *N*-methylimidazole gave the corresponding imidazolyl carbinol in excellent yields, Matsuura reported that the same substrate gave the corresponding carbinol in 9% yield. They also reported that 2-methylimidazole gave a mixture of two products with an overall yield of 22.5% [119]. 1,2-Dimethylimidazole reacted with benzophenone. However, this compound gave a reaction on the methyl in the 2 position in low yield [120,121]. *N*-Benzylimidazole, when irradiated in the presence of benzophenone in *t*-BuOH, gave a reaction on the methylene group [120,121]. Only the irradiation of *N*-acetylimidazole allowed to obtain the corresponding oxetane when the reaction was performed in the presence of benzophenone (Scheme 25). The same result was obtained by using *N*-benzoylimidazole and *N,N’*-carbonyldiimidazole [120,121].

The oxetane can be obtained also in the reaction of *N*-methyl-2,4,5-triphenylimidazole with aromatic ketones. It is noteworthy that, in this case, the reaction did not work in the presence of acetophenone and benzaldehyde [122].

The irradiation in the presence of benzophenone of 2,4-dimethylthiazole gave the corresponding oxetane, while the same reaction failed when acetophenone was used as carbonyl compound [123]. The irradiation in the presence of benzophenone of 3,5- and 4,5-dimethylisoxazole gave the corresponding oxetane in good yields, while 4-methylisothiazole gave a reaction on the methyl substituent [123].

**Scheme 26:**
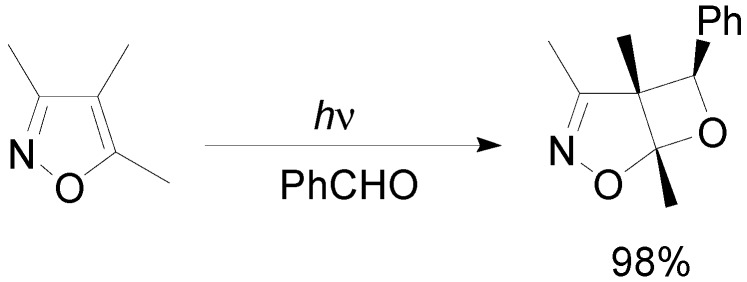
Reaction of an isoxazole derivative.

Recently, the reactivity of isoxazole derivatives has been revisited, showing that some derivatives, such as 3,4,5-trimethylisoxazole, gave the corresponding oxetanes in very good yield (in the case reported in the Scheme 26, almost quantitative yields were obtained) and excellent diastereoselectivity (>99:1) [124].

On the contrary, 3-phenyl substituted derivatives did not give the Paternò-Büchi reaction, allowing one to obtain only the ring contraction products [124]. In this case, the isoxazole was able to absorb the light giving the corresponding excited state able to give the isomerization reaction [125,126,127,128].

Aliphatic and aromatic carbonyl compounds reacted with oxazole derivatives with good to high exo diastereoselectivity, but low facial stereoselectivity. This reaction can be used in the synthesis of erythro-α-amino β-hydroxy carboxylic acid derivatives [129,130,131,132,133].

The irradiation of indole with benzophenone did not give the corresponding adduct. On the contrary, a benzoyl derivative of indole reacted with benzophenone, giving the corresponding oxetane [134]. It did not react with acetophenone, benzaldehyde, acetone, and propionaldehyde. When methyl pyruvate was used as carbonyl compound, the corresponding 3-indolyl carbinol was obtained [134].

The same reaction has been described on *N*-acetyl derivative of 7-azaindole. Although the reaction represents a method able to obtain a new class of compound, the low yields of the product (4%) prevent a synthetic use of this reaction [134].

**Scheme 27:**
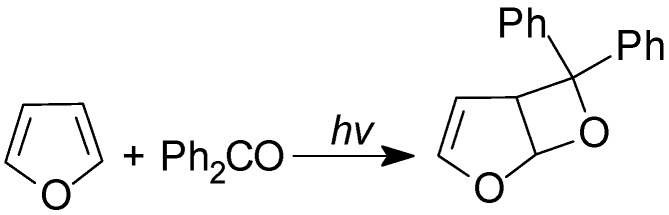
Reaction of furan.

Schenck reported that the irradiation of benzophenone in furan gave the corresponding adduct in 94% yield (Scheme 27) [135]. The structure was confirmed by Gagnaire *et al* [136].

Furan and 2-methylfuran were found to react with propanal and benzaldehyde [111,137,138,139,140,141,142]. In this case, the *exo* stereochemistry at C-6 on the dioxabicyclo[3.2.0]heptene skeleton has been assigned [143]. Good regioselectivity was observed using silyl and stannyl furan derivatives: in this case, the reaction occurred on the less hindered side of the molecule [144]. When 2-silyloxyfuran was irradiated in the presence of aliphatic carbonyl compounds or with benzaldehyde, it gave a 1:1 mixture of regioisomeric products. On the contrary, when benzophenone was used, only the product deriving from the attack on the most hindered side of the molecule was recovered. The same result was obtained using acetone in a reaction when a low concentration of furan was used. In all the cases, an *exo* selectivity was observed [145]. On the contrary, 2-furylmethanol and the corresponding silyl ether gave low regioselectivity [145,146].

**Figure 5:**
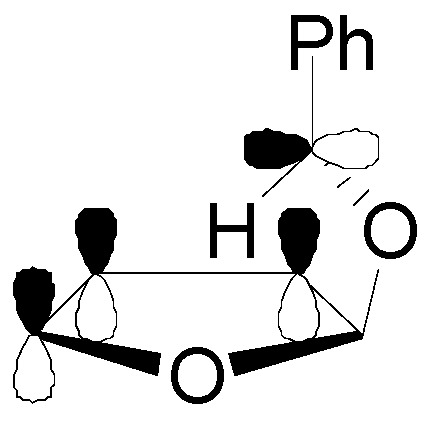
Structure of the biradical intermediate in the reaction of furan with benzaldehyde.

The high *exo* stereoselectivity of the reaction has been extensively studied: the formation of the product occurred on a triplet 1,4-biradical. The triplet biradical must be converted into the singlet one to give the product. In order to explain the pronounced *exo* stereoselectivity, a secondary orbital effect can be postulated: an interaction between the rather flexible α-oxy radical center and the allyloxy ring localized radical likely plays a major role (Figure 5) [147,148].

**Figure 6:**
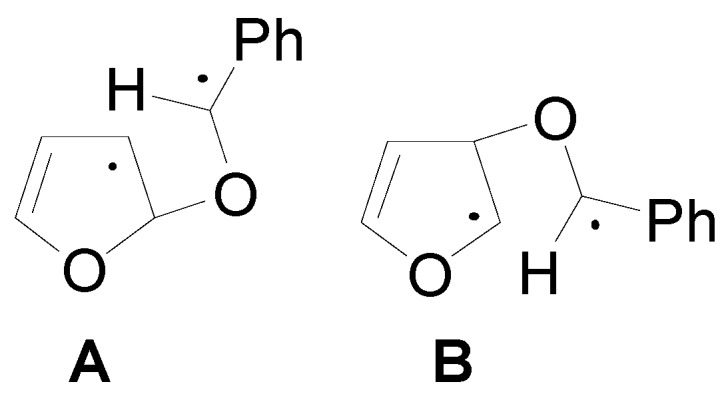
Possible biradical intermediates in the reaction of furan with benzaldehyde.

The regioisomeric biradical intermediates **A** and **B** resulting from the head-to-head and head-to-tail addition, respectively, have been examined (Figure 6) [69]. The biradical **A** is more stable than **B** by 16.5 kcal mol^−1^. The biradical **A** exists as two conformers and only that conformer able to give the ring closure was considered.

**Scheme 28:**
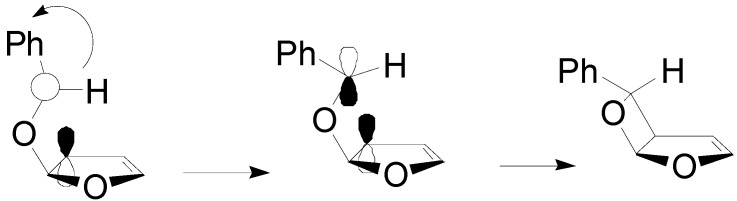
Ring closure reaction in the formation of the *exo* isomer of the adduct between furan and benzaldehyde.

The HSOMO is mainly localized on the benzaldehyde fragment of the biradical while the LSOMO is mainly localized on the furanoid part of the molecule. The coupling between the radical carbons in these two orbitals, considering that the atomic orbitals involved can superimpose themselves, can give only the *exo* isomer, in agreement with the experimental results (Scheme 28).

When the reaction was carried out in benzene, dimers can be obtained [55,149,150,151]. Furan reacted with chloral to give unexpectedly the corresponding 2-furyl carbinols [152]. Furthermore, 2-cyanofuran did not react, while 2-furfural diacetate and furfural ethylene acetal showed low reactivity [152].

**Scheme 29:**
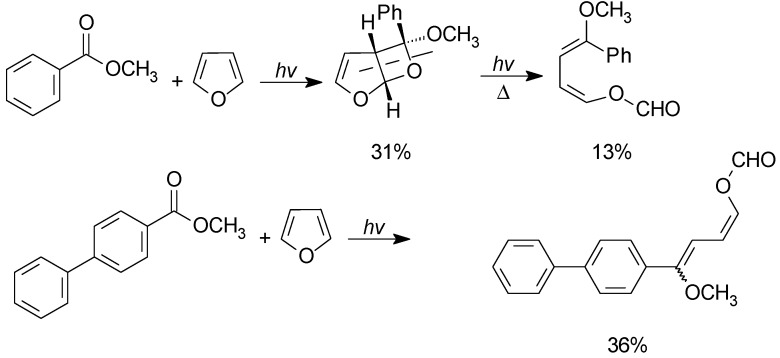
Metathesis reactions on oxetanes obtained from furan derivatives.

The cycloaddition reaction can be performed on esters. In this case, the adducts can be obtained in a few cases. In most of the examples, they underwent a cycloreversion reaction to give the ring opening products (Scheme 29) [153]. More recently, this result has been questioned and a 95:5 mixture of stereoisomeric adducts was identified as the product [148]. Cycloreversion products were obtained also in the reaction of furan in the presence of furan-2-carboxyaldehyde [154].

Coupling products can be obtained carrying out the reaction between an amide and furan: also in this case the cycloadduct cannot be isolated, but the subsequent decomposition products can be isolated [155]. Furan quenches the fluorescence of the substrate, while a small new emission at 500 nm appears: this evidence is in agreement with a mechanism involving a reaction in the excited singlet state *via* the formation of an exciplex.

The reaction of some aromatic carbonyl compounds (benzophenone, benzaldehyde) with benzofuran has also been reported; when compounds showing high triplet energy were used, dimers of benzofuran were obtained. On the contrary, when carbonyl carbonyl with a low triplet energy were used, oxetanes were the products of the reaction [156]. As in the case of furan, Schenck obtained only one regioisomer. A reinvestigation of the reaction of benzofuran when it was irradiated in the presence of acetophenone or propiophenone, showed that, also in this case, the corresponding oxetanes were the products of the reaction [157].

The irradiation of 4-amino-2,7-dimethylbenzofuran in the presence of benzaldehyde in benzene gave the corresponding adduct in good yields [158]. It is interesting to note that the reaction allowed the formation of the *endo* isomer. The reaction between benzo[*b*]furan and benzaldehyde gave an adduct whose stereochemistry has not been described [156].

3-Furylcarbinol derivatives could be obtained through the treatment of the oxetanes with TsOH [142]. The formation of a protonated oxetane is in agreement with the high negative entropy of activation [159]. If the irradiation was performed in the presence of an acid, 3-furylcarbinol can be obtained in a one-step procedure in better yields [160]. This procedure has been used in the synthesis of perillaketone, a naturally occurring 3-substituted furan [160]. On the contrary, Lewis acids catalyzed a different behavior: the treatment with BF_3_^.^Et_2_O gave only 3-substituted furan in THF and 89% of 2-substituted furan in acetonitrile [159].

The oxetane derivatives were treated also with KMnO_4_ and the resulting *cis* diol reacted with acetone in the presence of an acid. This procedure allowed the synthesis of a carbohydrate derivative [161].

An epimerisation reaction occurred on the *trans* diol obtained when the oxetane was treated with MCPBA [161]. Schreiber studied the possible chemical modifications on the cycloadduct deriving from the reaction between an aldehyde and furan [162]. Hydrolytic ring opening, reductions, hetero Diels-Alder, the reaction with MCPBA, and hydroboration-oxidation were the reactions he studied. The reaction with MCPBA was used by Schreiber and coworkers in the synthesis of asteltoxin [163,164], and in that of avenaciolide, an antifungal metabolite [165]. The reaction of tributylstannylfuran with butyl glyoxylate was used in the synthesis of a ginkgolide B-kadsurenone hybrid of two inhibitors of a platelet activating factor [144].

**Scheme 30:**
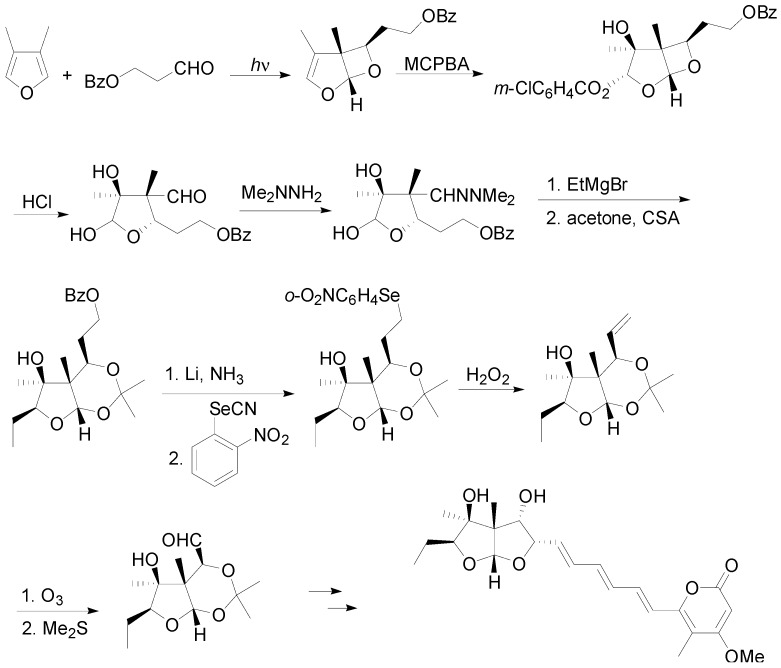
Synthesis of asteltoxin.

In the synthesis of asteltoxin, the synthetic sequence implies the photochemical coupling of 3,4-dimethylfuran with a functionalised aldehyde to give the corresponding adduct in 63% yield (Scheme 30).

The same research group reported a formal synthesis of avenaciolide, an antifungal metabolite. In this case the oxetane (obtained in multigram quantities in high yields and with complete stereochemical control) was treated with hydrogen to give the saturated compound. The key step in this synthetic procedure is a reaction with ozone followed by a base catalysed epimerisation with potassium carbonate and cyclization in acidic medium.

Furthermore, the cycloadduct obtained from the reaction between furan and an aldehyde can be treated with an excess of Schlosser’s base (BuLi, *t*-BuOK). The reaction gave the corresponding anion which can react with carbonyl compounds or alkyl halides [166].

The most important target in this field was oxetanocin (**3**), a nucleoside isolated from *Bacillus megaterium* NK 84-0218 showing anti-HIV activity. An approach to the synthesis of this compound has been reported [167]. The treatment of oxetane obtained in the reaction between furan and benzaldehyde with *N*-iodosuccinimmide in the presence of methallyl alcohol gave the corresponding iodoacetals. The subsequent treatment with iodonium di-*sym*-collidine perchlorate (IDCP) gave a product with a structure related to that of **3** in low yields as a relatively unstable mixture of diastereoisomers.

Oxetanocin was also obtained carrying out the reaction between 2-methylfuran and benzoyloxyacetaldehyde [168]. The corresponding adduct was treated with ozone and the product was reduced with NaBH_4_. The obtained alcohols were protected. The product was treated with N-benzoyl-disilyladenine and SnCl_4_ to give **3**.

**Scheme 31:**
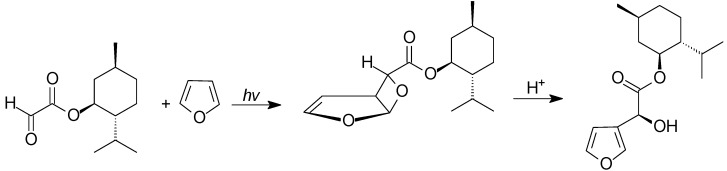
Reaction of furan with a chiral glyoxylate.

The reaction of glyoxylates with furan can be performed also using chiral glyoxylates. In particular, the use of *R*-(‒)-menthol, 2-octanol, and 2,2-dimethyl-3-butanol as chiral auxiliaries gave the corresponding oxetanes in high yields. These compounds can be converted into the corresponding 3-substituted furans. The furans showed low enantiomeric excess (Scheme 31) [169].

The use of chiral phenylglyoxylate gave better results. The use of chiral alcohols gave diastereoisomeric excess in the range of 4%–80% [170,171,172,173].

An important variability of the diastereoisomeric excess in function of the temperature has been observed with the presence of an inversion temperature [174]. When the reaction is carried out on 2-methylfuran, a 2:1 regioisomeric mixture was obtained with a very high diastereoisomeric excess [175].

**Figure 7:**
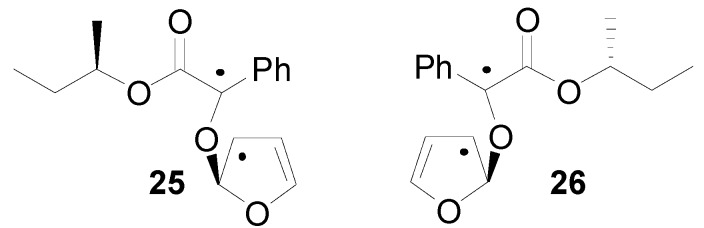
Radical intermediates in the reaction of chiral phenylglyoxylates with furan.

In order to justify the observed stereoselectivity the triplet biradical intermediates in the reaction of some phenylglyoxylate derivatives with furan could be considered [173]. Calculations on these biradical intermediates showed that **25** (the precursor of the observed product) was more stable than **26** by 0.73 kcal mol^−1^ (Figure 7).

Neckers studied the reaction of ethyl phenylglyoxylates with several alkenes showing that it gave the cycloaddition reaction only when electron-rich alkenes were used. Furthermore, the main reaction observed when monosubstituted alkenes were used was the Norrish type II reaction and the same behavior was observed reducing the electron richness of the alkenes [176]. Methyl 2-thienylglyoxylate showed a transient absorption at 390 nm and a broad band around 600 nm. This transient band has been assigned to the triplet state. This compound reacted with 2,3-dimethyl-2-butene to give the corresponding adduct [177].

**Scheme 32:**
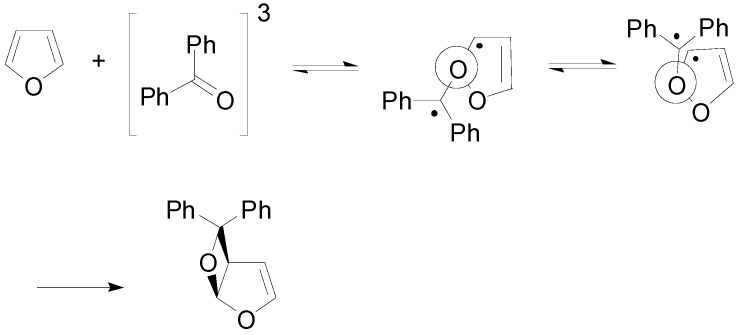
The reaction of furan with benzophenone. Conformers of the biradical intermediate.

To improve diastereoselectivity, the reaction was carried out in the presence of different zeolites [178]. NaY was the best solid zeolite support for this reaction. The origin of this behavior can be explained considering the possible effect of the confinement on the reaction. In the Paternò-Büchi reaction the biradical intermediate can assume two possible conformations [30,68]. One has the radical carbons in almost *anti* conformation, while the other one shows a *syn* conformation (Scheme 32).

The *syn* conformation can undergo the following cyclization, after the intersystem crossing from the triplet to the singlet state. For the *anti* conformation the most favored process is the retrocleavage to the starting material. The *syn* conformations take up a smaller volume than the *anti* one. The preferential formation of a stereoisomer in the reaction within a zeolite could be explained: the *SS-syn* conformer was obtained preferentially, while the *RS* conformer was obtained preferentially in *anti* conformation. The latter conformer has to rotate along the C-O bond to give the *syn* conformation able to cyclize. Zeolites could act as agents able to reduce the conformational mobility of the intermediates. They inhibited the rotation of this conformer and, then, favored the formation of the product deriving from the *SS* biradical intermediate.

Recently, Abe reported an attempt to obtain an intramolecular version of the Paternò-Büchi reaction between furan and a carbonyl compound. Nevertheless, the irradiation of a phenylglyoxylate derivative allowed only the formation of a product deriving from an intermolecular reaction. Low yields were obtained [179].

The irradiation of furan in the presence of acyl cyanides yielded the corresponding oxetanes but both diastereoisomeric *endo-* and *exo*-oxetanes are formed. Low asymmetric induction was observed when chiral acyl cyanides were used [180]. Furan reacted also with chiral ketones. In this case, before the 2+2 cycloaddition, an α-cleavage reaction modified the expected products. A chiral product was obtained as 2:1 diastereoisomeric mixture where the most abundant product has 1*R*, 3*R* configuration when (‒)-menthone was used as a substrate [181]. If the irradiation was performed on the ketonic functional group in a protected carbohydrate, a complex reaction mixture was obtained [182,183].

The reaction between 3,4-dimethylfuran and *R*-isopropylidene glyceraldehyde was performed in order to obtain a stereoselective Paternò-Büchi reaction. The coupling products were obtained with an overall yield of 35% as a 1.2:1 mixture of diastereoisomers [184].

This behavior suggested a mechanism that was insensitive to the substitution pattern of chiral aldehydes. Reaction between an excited aldehyde (singlet or triplet state) and furan proceeds with initial carbon-oxygen bond formation to produce either of the two biradical species. The stereocenter adjacent to the carbonyl is now in a 1,4-relationship to the newly formed stereocenter at the acetal carbon and is expected to exert little influence as a stereocontrol device [184]. This hypothesis is not in agreement with the stereochemical behavior of using phenyl glyoxylate esters where the chiral carbon atom was farther than in *R*-isopropylidene glyceraldehyde. The extensive racemization observed probably reflects the photolability of the aldehydes towards racemization under the conditions of the reaction [184]. Nevertheless, the product of this reaction was used in a chiral synthesis of the bicyclic part of asteltoxin confirming the assigned absolute configuration [184]. On the contrary, benzoin reacted with furan to give the corresponding adduct in acceptable yield (56%) and *de* > 98% [185].

The observed stereoselectivity was explained considering the relative stability of the biradical intermediates. Nevertheless, chiral ketones gave the Norrish Type II reaction as the only observed reaction. In order to avoid Norrish Type II reaction a substrate without γ-hydrogen was used [186].

**Scheme 33:**
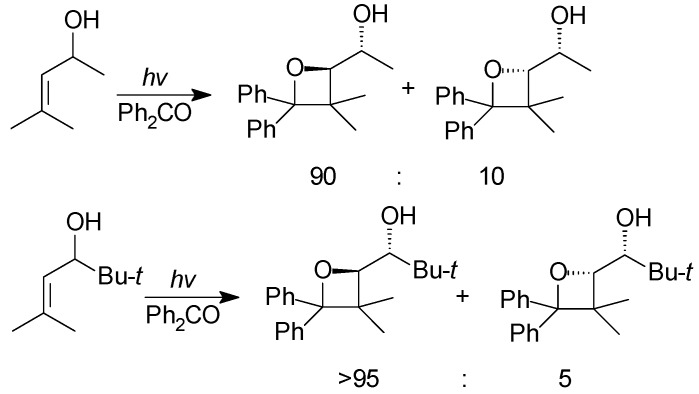
Reaction of allylic alcohol derivatives with benzophenone.

In 1990 Griesbeck found that the reaction of benzaldehyde with homoallylic alcohols did not show diastereoselectivity [61]. Nevertheless, Adam showed that allylic alcohols reacted with benzophenone to give the corresponding adducts with high regio- and diastereo-selectivity (Scheme 33) [187,188,189,190].

In presence of protic methanol, the diastereoselectivity dropped drastically, while it totally disappeared using the corresponding silyl ethers. These data are in agreement with the presence of a hydroxyl directing effect in the Paternò-Büchi reaction. The formation of a hydrogen bond between triplet excited benzophenone and the substrate in the exciplex favored the formation of the *threo* isomer. On the contrary, the formation of the *erythro* stereoisomer would be less favored due to allylic strain.

The formation of a hydrogen bond to direct the Paternò-Büchi reaction has been considered by other researchers. Diastereoselective cycloaddition has been obtained using chiral enamide [191,192], or in the reaction of allylic alcohols with naphthalene rings [193]. When unsymmetrical carbonyl partners such as acetophenone or benzaldehyde were used, the corresponding *cis* isomer was observed with high diastereoselectivity. The regioselectivity was high with acetophenone but lower with benzaldehyde [188].

*Cis* diastereoselectivity can be explained by using the Griesbeck rule on the possible triplet biradicals formed in the reaction. Steric interactions are minimized when the biradical assumes the optimal conformation and this conformation accounts for the formation of the observed stereoisomer [190].

When chiral allylic alcohols were used as substrates in the reaction *cis* diastereoisomers were formed. Furthermore, also in this case, a pronounced *threo* diastereoselectivity was observed, in agreement with a less pronounced hydroxyl directing effect when acetophenone and benzaldehyde were used [188,190]. Chiral allyl ether gave the corresponding adduct with high diastereoselectivity [98]. We have to note that this result is not in agreement with previous reported data on the reactivity of silyl ethers of allylic alcohols [187].

The reaction of 2,3-dihydrofuran-3-ol derivatives (a particular type of allylic alcohol) with benzophenone gave the corresponding adducts. In methanol, a *trans* relationship between the oxetane ring and the hydroxyl group was observed, while, in benzene, the *cis* isomer prevailed. The Eyring plot showed that the *trans* isomer increased with a not linear behavior upon decreasing the temperature [194].

**Scheme 34:**
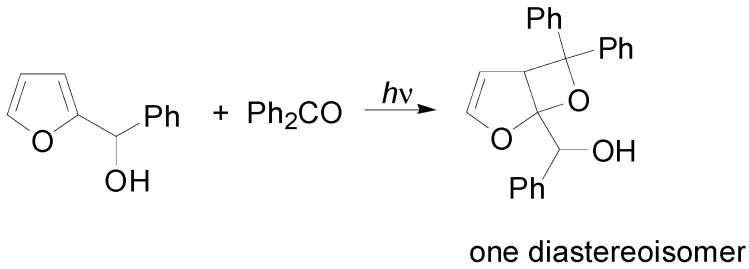
Reaction of 2-furylmethanol derivatives.

The reaction of allylic alcohols with carbonyl compounds was tested also on 2-furylmethanol derivatives. The presence of large substituents on the carbon bearing the alcoholic function allows a high regioselectivity (Scheme 34) [195].

5-Methyl-2-furyl derivatives were used as substrates showing a different regioselectivity. This type of substrates gave a 1:1 mixture of regioisomers, when irradiated in the presence of benzophenone, and a single regioisomer in the presence of benzaldehyde [196]. In agreement with the results obtained with 2-furyl derivatives, the products deriving from the attack on the side bearing the alcoholic function were obtained as a single diastereoisomer, while those deriving from the attack on the side bearing the methyl group were obtained as a mixture of diastereoisomers.

The above described reactions showed that on furan two possible regioisomers could be obtained. The experimental results showed that, in some cases, the reactions occurred mainly on the side when the hydroxyl group was present, while other reactions showed a completely different regioselectivity. The reason of this behavior can be found in kinetic factors depending on the different stability of the biradical intermediates. These considerations are in agreement with the results obtained irradiating geraniol in the presence of benzophenone [197].

In this case, two different double bonds were present: a double bond able to give a tertiary radical intermediate, and an allylic alcoholic moiety. The authors found that at −75 °C the product deriving from the attack on the first double bond was obtained in 44% yields while the product deriving from the attack on the allylic double bond was obtained only in 13% yields. Furthermore, at 20 °C the yields were 29% and 15%, respectively. In this case, then, the attack on the terminal double bond was favored.

The reaction of 2-furylmethanol derivatives with aliphatic aldehydes and ketones gave the corresponding adducts with high regioselectivity: the reaction occurred on the most hindered side of the substrate. However, no diastereoselectivity was observed [198]. The relative stability of the biradical intermediates was able to explain the regioselectivity of the reaction. A computational study (DFT) showed that the biradical obtained on the most hindered side of the molecule was more stable than the other one [196].

The diastereoselectivity of the reaction between 2-furylmethanol derivatives and aromatic carbonyl compounds clearly showed that it increased in relation to the nature of the substituents on the carbon bearing the alcoholic function as described by Adam. However, while Adam considered the allylic strain with a methyl group in β-position as the driving force for the diastereoselectivity, in this case, a methyl group on the C-3 of the furan ring was not present.

In order to have more data to explain the observed stereoselectivity the photochemical behavior of tertiary 2-furylcarbinols was studied [199]. The irradiation of 1-methyl-1-phenyl-1-(2-furyl)methanol with benzaldehyde gave a mixture of two regioisomeric products. The regioisomer on the most hindered side of the molecule was obtained in low yield but it showed a complete diastereoisomeric control. On the contrary the main product was a mixture of four diastereoisomeric products. The reaction of the same compound with benzophenone gave only the product deriving from the attack on the most hindered side of molecule. This compound was obtained with 48% diastereoisomeric excess.

The regioselectivity was explained, as described above, considering the relative stability of the biradical intermediates. In this case, in the reaction of the 1-methyl-1-phenyl-1-(2-furyl)methanol with benzaldehyde the biradical obtained on the less hindered side of the substrate was more stable than the other one by 18.03 kJ mol^-1^. Recently, some authors reported that no stereoselectivity was observed using cyclic 2-furyl methanol derivatives [200]. However, in another work, a stereoselective behavior of the same substrates was assumed [201].

**Scheme 35:**
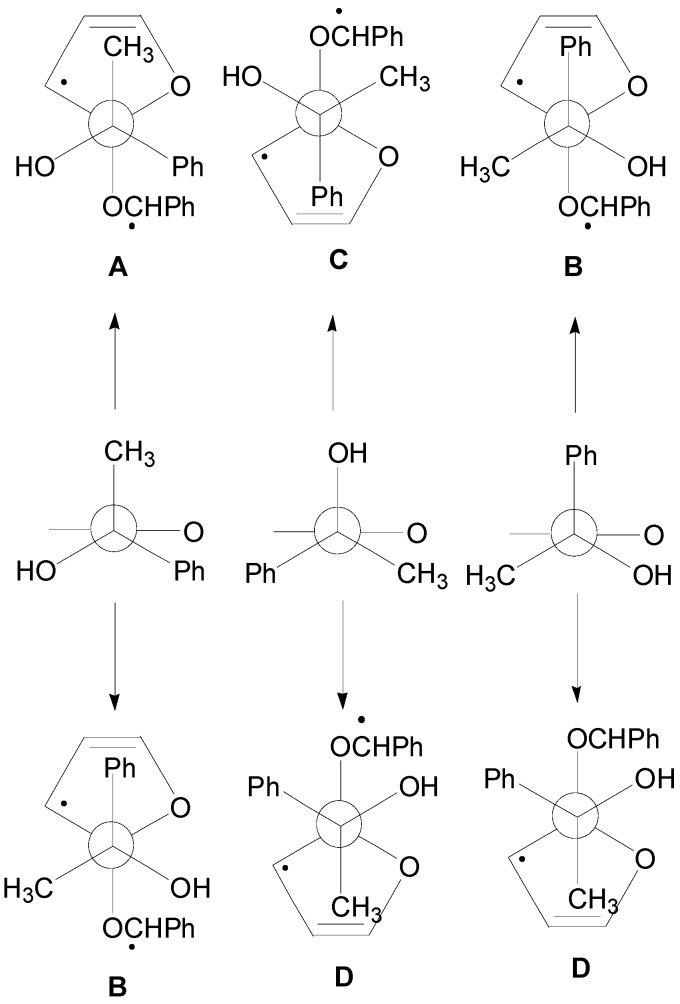
Possible conformations of the biradical intermediate from the reaction of 1-methyl-1-phenyl-1-(2-furyl)methanol with benzaldehyde.

On the basis of these results an explanation of the stereochemical behavior was attempted [199]. 1-Methyl-1-phenyl-1-(2-furyl)methanol showed three conformations. All three conformers were in the range of 1.97 kJ mol^-1^ and they did not show a preference. The directing effect exerted by the hydroxyl group is due to the formation of a hydrogen bond between the hydroxyl group and the oxygen of the excited carbonyl compound, or it is due to the formation of a complex. This type of interaction could favor the formation of a preferential conformation in the biradical intermediate where the hydroxyl group and the oxygen of the carbonyl compound are near. These conformations could have different energies for different diastereoisomeric biradicals, giving an explanation of the observed behavior. In the case of 1-methyl-1-phenyl-1-(2-furyl)methanol, if the hydroxyl group drove the attack of the oxygen of the carbonyl group, the conformations of the biradical intermediate represented in Scheme 35 were obtained. **B** and **D** were the preferential conformations: calculations on these conformations showed that there was a difference of 13.26 kJ mol^−1^ between the energies of these two conformations. This difference can account for the observed complete diastereoselectivity of the reaction. In the reaction of the same substrate with benzophenone, the corresponding conformers **B** and **D** showed a difference energy of 7.79 kJ mol^−1^: this difference is in agreement with the observed diastereoselectivity.

The same approach can be used to justify the stereochemical behavior of the reaction of allylic alcohols with benzophenone [202]. The irradiation of 3-furylmethanol derivatives in the presence of benzophenone gave the corresponding adduct with a very high stereoselectivity [203].

The above reported results represent all the available data on the Paternò-Büchi reaction on pentaatomic heterocycles. We can see that, with the exception of furan, there are very few data: in particular, 1. most of the unsubstituted tested compounds different from furan did not react, 2. only few substituted derivatives showed a significative reactivity towards excited carbonyl compounds.

This behavior may be due to different reasons. First, the different aromaticity of the compounds could play an important role in order to define the reactivity of the compounds. Furan is the lowest aromatic pentaatomic heterocyclic compound known, while the other compounds show higher aromaticity. However, this type of explanation cannot justify why thiophene does not react while simple dimethylthienyl derivatives react and why some dimethylthienyl derivatives react while some others do not show any reactivity.

A different explanation was identified in the quenching properties of these heterocycles. Thiophene and monomethyl derivatives are efficient quenchers of triplet benzophenone. The Stern-Volmer plot showed a linear relationship [204,205]. On the contrary, 2,5-dimethylthiophene (a compound able to give the cycloaddition reaction) is not a good quencher of the triplet benzophenone [206]. In this case, the Stern-Volmer plot is not linear. This situation is commonly encountered when the quencher employed quenches two excited states. It seems reasonable that pyrrole acts as quencher of both triplet benzophenone and the exciplex between triplet benzophenone and pyrrole. 

On the basis of these investigations, the common five-membered heterocycles may be classified in two categories in regard to their quenching properties: those with electron-donating groups, which give Stern-Volmer plots in the shape of straight lines, and those substituted with halogens and electron attracting groups, such as *N*-benzoylpyrrole, which give parabola-shaped curves. Those in the first category give oxetanes when they are bad quenchers. Since these compounds do not presumably form exciplexes, they should go from starting materials to products through a biradical intermediate. On the other hand, those in the second category most likely give oxetanes when they are good quenchers, as may be shown for 2,5-dibromothiophene, and furthermore they go from starting materials to products through an exciplex [207].

**Scheme 36:**
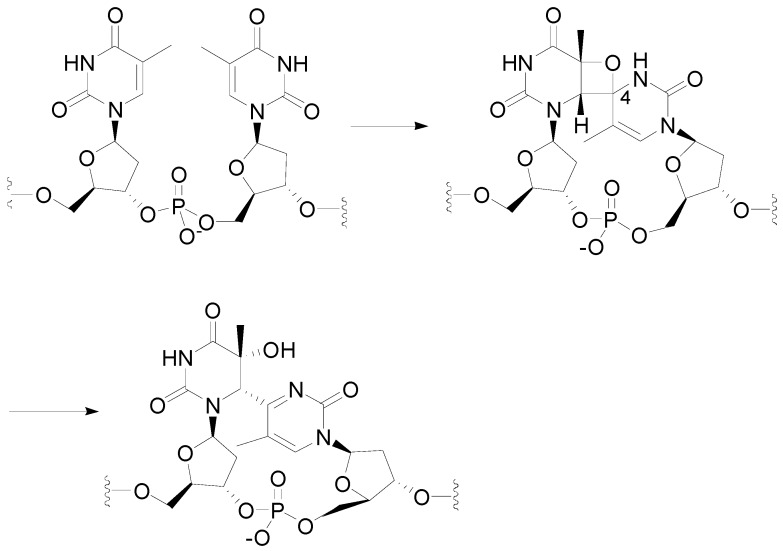
Photochemical reaction between DNA pyrimidine derivatives.

The (6-4) photoproduct is an adduct of two pyrimidines that occupy adjacent sites on the same DNA strand. It is the second major lesion induced in DNA by UV radiation. Although statistically four times less frequent than CPD lesion, (6-4) photoproducts are believed to be severely mutagenic. Its formation is believed to occur via an initial Paternò-Büchi type cycloaddition to form an oxetane intermediate. Subsequent C4-O bond cleavage gave the observed (6-4) photoproducts (Scheme 36).

**Scheme 37:**
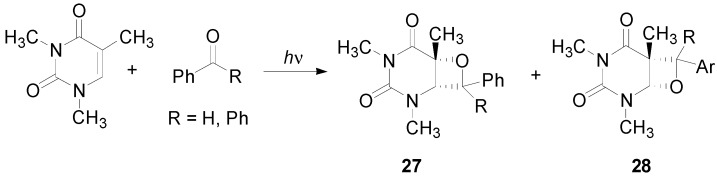
Reaction of uracil derivative with benzophenone and benzaldehyde.

The (6-4) photoproduct is one of the major mutagenic classes of DNA photoproducts and is involved in the etiology of skin cancer. The oxetane **27** was prepared in a triplet reaction and both electron donors and acceptors substituents were found to be able to photosensitize the splitting reaction (Scheme 37) [208,209,210,211]. Only one regioisomer (**27**) was observed. The other regioisomer **28** was rarely observed [212].

The temperature can determine the regiochemistry of the reaction. At −38 °C the regioisomer **27** dominates (61:39). On the other hand, at 70 °C the regioisomeric behavior of the reaction is inverted [212,213]. A change of the selectivity-determining step has been determined considering a non-linear Eyring plot. Two different cases can be assumed: the situation where the conformational changes of the triplet intermediates were slower than ISC at low temperature and the case where the conformational changes exceed the ISC process [213]. When the conformational interchange is faster than the ISC process, the population of high potential energy conformations decreases, while the population of a lower potential energy conformer increases.

A weak solvent effect was observed on the regioselectivity of the reaction [214]. Substituents on benzophenone modified the regiochemistry of the reaction [215,216,217]. The quantum yields correlated with the energy gap between SOMO of benzophenone derivatives and the HOMO of the uracil. Recently, a computational approach to this reaction appeared [218]. The authors sudied the biradical intermediates. They found that one of them is more stable than the other, and that the formation of the first is faster than the other one. Then, **28** can be considered as a thermodynamic product, while **27** a kinetic one.

**Scheme 38:**
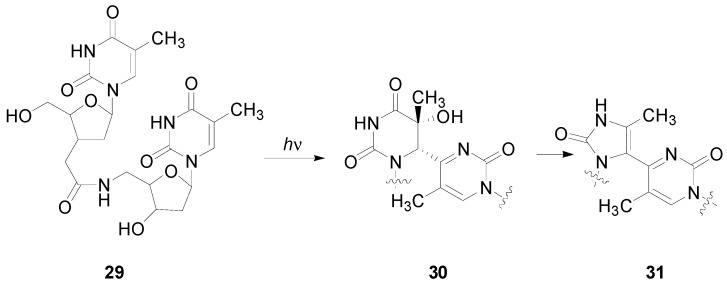
Irradiation of **29**.

Recently, it has been observed that, due to the energy barriers between the two stable conformers, the equilibrium was more favorable for the formation of the oxetane **28**, rather than oxetane **27** at a higher temperature. Triplet benzophenones with a short lifetime would give rise to a less efficient Paternò-Büchi reaction [217]. The oxetane was obtained also in the reaction between benzophenone and benzophenone-derived drugs and thymidine [211,219]. An enantioselectivity factor for triplet deactivation was found using enantiopure ketoprofen. The use of enantiopure ketoprofen showed that thimidine was able to give an enantioselective quenching of the chiral ketoprofen triplet state. This quenching was related to the formation of C-O bond, the first step of oxetane formation [67]. An intramolecular reaction beween thymidine esterified by ketoprofen has been reported [220]. The irradiation of **29** gave **30** deriving from the (6–4) photoadduct **31** (Scheme 38) [221].

A reaction between 5-fluoro-1,3-dimethyluracil with 1,5-dimethoxynaphthalene has been reported [222]. The reaction gave product where an aromatic ring of naphthalene is broken. The presence of the intermediate adduct has been proposed.

## 5. Other intermolecular reactions

**Scheme 39:**
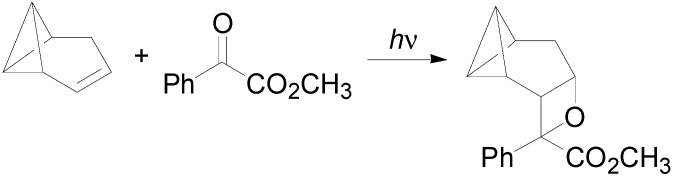
Reaction of homobenzvalene.

5-Substituted adamantan-2-ones gave the corresponding cycloadducts via the n, π* excited singlet state. This substrate gave a regiospecific reaction with a low stereoselectivity (the best result was 59:41 ratio in favor of the *anti* isomer) [223,224,225,226]. Methyleneadamantane reacted with acetone. However, the Paternò-Büchi adduct was obtained in only 5% yield [223]. The reaction of biacetyl with benzvalene gave the corresponding adduct, while benzophenone gave, as the only product, benzene [44]. When homobenzvalene was used as the alkene in the reaction with ethyl phenylglyoxylate, the irradiation gave the corresponding adduct in 70% yield with high stereoselectivity (Scheme 39) [227].

**Scheme 40:**
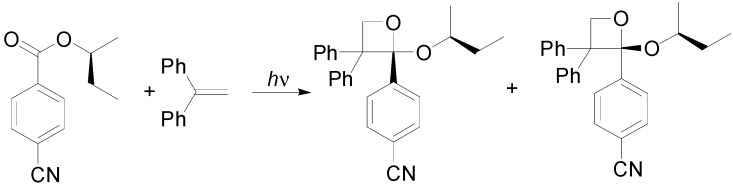
Esters as carbonyl compounds in the Paternò-Büchi reaction.

Esters could be used as carbonyl compounds in the reaction with chiral alkyl cyanobenzoate derivatives (Scheme 40) [228,229].

The reaction occurred through the first excited singlet state. The authors showed the presence of a charge transfer band at 300–330 nm. Irradiating at 254–290 nm, a good diastereoselectivity was observed (*de* 77%). The irradiation at 330 nm afforded the formation of an epimeric mixture. The photocyclization was likely to proceed *via* a short lived 1,4-biradical mechanism. Then, the diastereo-face-differentiating complexation of chiral alkyl cyanobenzoate with the olefin donor is the responsible for the observed *de* values. Calculations showed that the conformational population in the charge-transfer complex is almost the same in agreement with the observed lack in diastereoselectivity.

A stereoselective behavior has been described in the reaction between benzophenone and cyclooctene. At −95 °C the *cis* adduct was formed with a very high diastereoselectiviy from *cis*-cyclooctene. On the contrary, at −20 °C both diastereoisomers were generated. At higher temperatures, the *trans* adduct dominated. Over a broad temperature range (−80 to +60 °C), the more strained *trans*-cyclooctene gave nearly exclusively the *trans* adduct [93,94]. Triplet ketone could attack the double bond to give two biradical intermediates and the authors suggested that temperature-dependent conformational changes of the biradical competed with the cyclization to the oxetane and the retro cleavage to the *cis*-cyclooctene. The activation barrier between two conformers of the biradical intermediate could explain the increasing of *trans*-oxetane with the temperature [94].

1-Acetylisatin reacted with styrene and furan derivatives to give the *endo* adduct with high stereoselectivity [230,231].

**Scheme 41:**
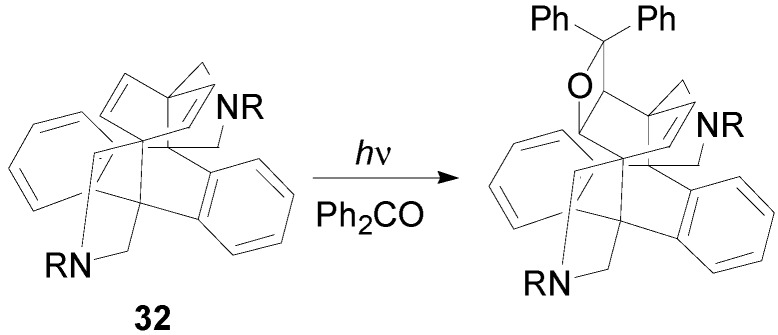
Reaction of a paracyclophane derivative.

Quinones react with stilbene derivatives has been described [73,232]. In this case, a charge-transfer absorption band was observed at 480 nm. Time-resolved (ps) absorption spectrum showed an absorption at 480 nm (chloranil anion radical) and a broad band around 760 nm. After 50 ps a transient absorption typical for triplet chloranil at 510 nm was observed. The reaction occurred through selective excitation at 480 nm. Quinones reacted with diarylacetylene giving a product deriving from the ring opening of a transient oxetene. Time-resolved (ps) absorption spectrum was in agreement with an electron transfer mechanism [233]. When 2-chloro-5-methoxybenzoquinone reacted with arylacetylenes, CIDNP experiments suggested a mechanism where the attack of the triplet quinone on the acetylene gives a biradical intermediate [92]. The same behavior has been observed in the reaction between quinone and quadricyclane and norbornadiene [234,235]. The adduct was found also in the reaction between quinones and acenaphthylene [77]. Benzophenone can react also with a paracyclophane derivative **32** (Scheme 41) [54].

The Paternò-Büchi reaction can also be applied to other unsaturated systems other than alkenes, such as dienes [10], allenes [236], acetylenes [237], and ketenimines [236]. When tetramethylallene reacted with aromatic ketones the main products resulted from bis-addition of the carbonyl compounds to give dioxaspiroheptanes [238,239]. On the contrary, 1,1-dimethylallene gave only the monoaddition 2:1 mixture of the adducts in a reaction with aliphatic aldehydes [240]. A higher regioselectivity was obtained using an allene derivative.

The irradiation of isoquinoline-1,3,4-trione with alkynes gave a product deriving from an initial Paternò-Büchi reaction on the carbonyl at C-4 to give the corresponding oxetene [241]. DFT calculations on the relative stability of the biradical intermediate showed that the more stable one was the precursor of the main product of the reaction.

## 6. Intramolecular Reactions

**Scheme 42:**
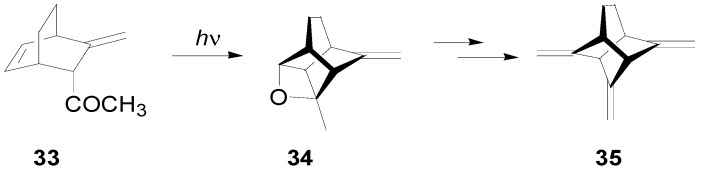
Intramolecular oxetane formation in the synthesis of 2,7,9-trimethylenetricyclo[4.3.0.0^3,8^]nonane.

An intramolecular Paternò-Büchi reaction on a paracyclophane derivative has been reported giving the corresponding adduct in quantitative yield [90]. An intramolecular Paternò-Büchi reaction of **33** to give **34** has been used in the synthesis of 2,7,9-trimethylenetricyclo[4.3.0.0^3,8^]nonane **35** (Scheme 42) [242].

The same synthetic scheme was used in the synthesis of 2,7,9-trimethylenetricyclo[4.3.0.0^3,8^]non-4-ene [243]. The preparation of some stelladiones such as tricyclo[3.3.0.0^3,7^]octane-2,4-dione or tricyclo[3.3.0.0^3,7^]-octane-2,6-dione [244]. This type of intramolecular reaction was the key step used in the synthesis of diquinanes and triquinanes [245,246,247].

The synthesis of 1,13-herbertenediol was performed using an intramolecular Paternò-Büchi reaction between an aldehydic group and an α-alkyl substituted styrene moiety [248]. An intramolecular stereoselective Paternò-Büchi reaction was the key step also in the synthesis of some derivatives of *R*-(+)-sclareolide [249]. A synthesis of the scaffold of merrilactone A involved also an intramolecular 2+2 cycloaddition to give the adduct [250].

On the other hand, a project devoted to obtain tromboxane analogs using an intramoelcular reaction of a carbonyl group with an enol ether, failed [251].

**Scheme 43:**
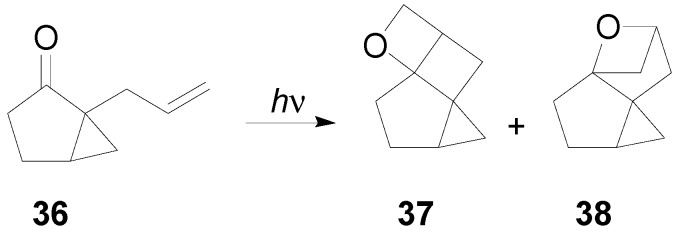
Intramolecular oxetane formation on allyl cyclopentanone derivatives.

An intramolecular reaction on allyl cyclopentanone derivatives has been reported [227,252,253]. In this case both *straight* **37** and *crossed* **38** oxetanes can be obtained (Scheme 43). Using 2-allylcyclopentanone nearly equal amounts of these isomers were obtained. However, the use of the more rigid starting material **36** allowed the preferential formation the *straight* isomer.

**Scheme 44:**
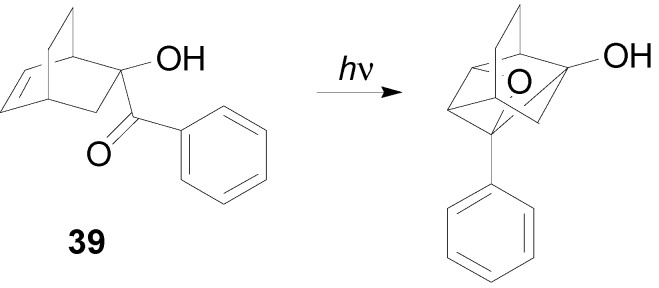
Intramolecular oxetane formation in the reaction of **39**.

An intramolecular cycloaddition reaction was found in the reaction of **39** (Scheme 44) [254].

**Scheme 45:**
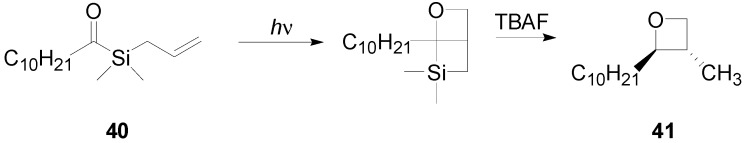
The use of silyl derivatives in the intramolecular synthesis of oxetanes.

Compound **40** gave in quantitative yield the oxetane through an intramolecular reaction. The oxetane thus obtained could be converted into **41** via fluoride desilylation (Scheme 45) [255].

When alkenyl phenylglyoxylates were used as substrates, Norrish type II is the main reaction in most of the cases [256,257].

*N-*Isopropyl-*N*-tigloylbenzoylformamides gave an intramolecular cycloaddition reaction in high yields (50-99%) to give the *syn* adduct (*syn/anti* = 2.1) [258,259].

**Scheme 46:**
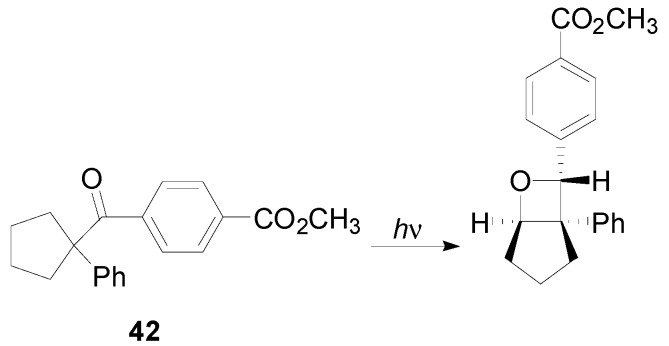
Chiral intramolecular oxetane synthesis.

When the ketone **42** was irradiated in the solid state, the only possible reaction was a Norrish Type I reaction. The reaction gave an alkene and a carbonyl compounds, able to react in an intermolecular reaction giving the corresponding adduct (Scheme 46). The reaction occurred with high stereoselectivity, and, when performed on a chiral salt gave the product with *ee* in the range 42%–52% [234].

## 7. The Future of the Paternò-Büchi Reaction

One of the most important field to be developed in the near future to obtain a more sustainable organic chemistry is connected to the flow chemistry. Several attempts have been made to apply flow techniques to photochemical reactions [260]. The photochemical reaction of benzophenone with prenyl alcohol to give the corresponding oxetane in flux conditions has been attempted with success [261].

Another field to be developed in the future is the use of photochemical reactions to functionalize polymeric materials. On this subject, two interesting results has been obtained. Oxetanes were obtained by using a photochemical coupling of aromatic aldehydes on a polymeric materials with alkenes [262]. On the other hand, also the coupling of polyvinyl compounds with aromatic aldehydes has been performed [263].

## 8. Conclusions

The aim of this review was to furnish an overview of the latest results obtained by using the Paternò-Büchi reaction. The reaction can be used as a suitable synthetic method to obtain an uncommon structural moiety such as the oxetane ring. The oxetane can be modified allowing the synthesis of several biological active compounds. Furthermore, the reaction can be accomplished in a stereoselective manner, allowing new sustainable chemical processes.
